# The association of HBV core promoter double mutations (A1762T and G1764A) with viral load differs between HBeAg positive and anti-HBe positive individuals: A longitudinal analysis^☆^

**DOI:** 10.1016/j.jhep.2008.09.014

**Published:** 2009-02

**Authors:** Zhong-Liao Fang, Caroline A Sabin, Bai-Qing Dong, Shao-Chao Wei, Qin-Yan Chen, Kong-Xiong Fang, Jin-Ye Yang, Xue-Yan Wang, Tim J. Harrison

**Affiliations:** 1 Division of Medicine, UCL Medical School, Windeyer Building, Cleveland Street, London W1T 4JF, UK; 2 Research Department of Infection and Population Health, Division of Population Health, UCL Medical School, Royal Free Campus, Rowland Hill Street, London NW3 2PF, UK; 3 Guangxi Zhuang Autonomous Region Center for Disease Prevention and Control, Jin Zhou Road, Nanning, Guangxi, PR China; 4 Sanitary and Antiepidemic Station of Long An, ChengXi Rd, Cheng Xiang Town, Long An, Guangxi, PR China

**Keywords:** BCP, basal core promoter, ORF, open reading frame, AFP, alpha fetoprotein, Hepatitis B virus, Basal core promoter double mutations, Viral loads, Hepatitis B e antigen (HBeAg), Anti-HBe

## Abstract

**Background/Aims:**

Although there have been a few reports regarding the effect of basal core promoter (BCP) double mutations (A1762T and G1764A) on hepatitis B viral loads, the association remains uncertain. We aim to determine the association after controlling for HBeAg – a strong confounding factor.

**Methods:**

We selected randomly 190 individuals from a Chinese cohort of 2258 subjects for cross-sectional analysis and 56 of the 190 for longitudinal analysis of viral loads.

**Results:**

In multivariable analysis of the cross-sectional data, BCP double mutations are significantly associated with lower viral loads in HBeAg positive subjects but no difference was found in anti-HBe positive subjects. Triple mutations at nucleotide (nt) 1753, 1762 and 1764 and mutations between nt 1809 and 1817, precore stop mutation (nt 1896) and genotype are not associated with viral loads in either HBeAg or anti-HBe positive subjects. Analysis of the longitudinal data yielded similar results to the cross-sectional data. Viral loads differ significantly between individuals infected with wild-type and BCP double mutations prior to HBeAg seroconversion but this difference is lost after seroconversion.

**Conclusions:**

BCP double mutations are associated with lower viral loads in HBeAg positive individuals but have no effect on the viral loads of anti-HBe positive individuals.

## 1 Introduction

Hepatitis B virus (HBV) has a 3.2 kb circular DNA genome containing four partially overlapping open reading frames (ORFs): C, encoding the nucleocapsid (core) protein (HBcAg) and secreted e antigen (HBeAg); P, the polymerase protein (Pol); S, the envelope proteins; and X, a transcriptional *trans*-activator protein. The core promoter located at nucleotides (nt) 1591–1822 plays a central role in virus replication, directing the synthesis of the pregenomic RNA, which, as well as being the template for genome synthesis, encodes HBcAg and Pol, and the precore RNA, which encodes HBeAg [1]. The basal core promoter (BCP) has been mapped to nt 1744–1804 [2].

The lack of proof-reading during reverse transcription of the pregenomic RNA favours the development of sequence variants during long-term HBV replication [3]. One of the most critical changes is the appearance of double mutations at nt 1762 (A–T) and 1764 (G–A) in the BCP. The mutations were first reported by Okamoto et al. who suggested that they might arrest the transcription of the precore RNA but not seriously affect that of the pregenomic RNA [4]. However, others suggested that these mutations seem to be insufficient to generate a HBeAg-negative phenotype [5] but may suppress the expression of HBeAg [6]. Subsequently, studies involving transfection of human hepatoma cell lines and analysis of clinical samples showed that these double mutations suppress, but do not abolish, the synthesis of HBeAg and may also and increase HBV DNA replication [2,7,8].

However, other reports do not support the conclusion that BCP double mutations increase virus replication, in that the BCP double mutations seem to have no effect on viral load [9–12] or even may be associated with lower serum HBV DNA concentrations [13]. Furthermore, others found in transfection studies that core promoter mutations other than those at nt 1762/1764 seem to upregulate viral DNA replication [14,15]. Therefore, the effect of the BCP double mutations on viral loads remains uncertain.

HBeAg has been used as a marker of infectivity and active virus replication in HBsAg-positive individuals [16,17]. Seroconversion from HBeAg to anti-HBe, either spontaneous or after antiviral therapy, usually results in lower viral loads, this decline occurring usually up to 12 months before seroconversion [18]. Furthermore, because of the complexity of the host immune system, viral loads may fluctuate over time [19]. Therefore, a single measurement, or several measurements made around the time of clearance of HBeAg, may not give a clear view of the effect of BCP double mutations on viral loads. In this study, taking the advantage of our Chinese cohort [20], we have carried out a cross-sectional analysis to determine the association of BCP double mutations and viral loads and a 3 year-longitudinal analysis to test this association further.

## 2 Study subjects and methods

### 2.1 The Long An cohort

In order to determine the value of screening carriers of hepatitis B surface antigen (HBsAg) for virus with BCP double mutations as a marker of an extremely high risk of developing HCC, a cohort of 2258 hepatitis B surface antigen positive subjects aged 30–55 was recruited in Guangxi, China. Written informed consent was obtained from each individual. The study protocol conformed to the ethical guidelines of the 1975 declaration of Helsinki and has been approved by the Guangxi Institutional Review Board and the UCL Committee on the Ethics of Non-NHS Human Research (Project no. 0042/001) [20].

From 1st March, 2004, the Chinese study team travelled to 128 villages in 12 townships of Long An county to visit agricultural workers aged 30–55 for a 3 ml sample of blood by venepuncture for screening for HBsAg. All samples were tested for HBsAg and those positive were tested in China for HBV DNA using nested PCR. We also detected and excluded those positive for anti-HCV or alpha-fetoprotein (AFP) to eliminate the confounding effect of HCV infection on the incidence of HCC and pre-existing HCC. We started to follow up our study subjects from 1st July, 2004. Each study subject completed a one-page questionnaire at the first visit and provided a serum sample every six months for the assessment of virological parameters and AFP concentrations and was monitored for HCC by ultrasonography (US).

### 2.2 Study subjects

#### 2.2.1 Cross-sectional analysis

We selected randomly approximately 25 subjects within each of eight sub-groups defined by the various combinations of sex (male or female), HBV status (HBeAg positive or anti-HBe positive) and BCP sequence (wild-type or 1762/1762 double mutations) (Table 1) [20].

#### 2.2.2 Longitudinal analysis

From these eight groups, we selected 58 study subjects for longitudinal analysis. Eligible subjects were those who did not convert from HBeAg to anti-HBe or from anti-HBe to HBeAg, whose HBV sequences remained stable at nt 1762, 1764 and 1896 over the 3 years, and for whom additional samples were available at each of three time points (12, 24 and 36 months). We also selected subjects for the analysis of occurrences of seroconversion and sequence evolution, and the association of these events with viral loads, including 10 individuals with evolution from wild-type BCP to double mutations but without HBeAg seroconversion and 17 individuals with clearance of HBeAg without evolution of the BCP. No subject received antiviral or immunosuppressive therapy during the 3-year period.

### 2.3 Serological testing

Sera were tested for HBsAg, HBeAg/anti-HBe using enzyme immunoassays (Zhong Shan Biological Technology Company, Limited, Guangzhou, China). Alanine aminotransferase (ALT) levels were determined using a Reitman kit (Sichuan Mike Scientific Technology Company, Limited, Chengdu, China).

### 2.4 Nested PCR for HBV DNA and nucleotide sequencing

DNA was extracted from 85 μl serum by pronase digestion followed by phenol/chloroform extraction. For nested PCR of the core promoter, first round PCR was carried out in a 50 μl reaction using primers B935 (nt 1240–1260, 5′-GAAGGTTTGTGGCTCCTCTG-3′) and MDC1 (nt 2304–2324, 5′-TTGATAAGATAGGGGCATTTG-3′), with 5 min hot start followed by 30 cycles of 94 °C for 30 s, 50 °C for 30 s, and 72 °C for 90 s. Second round PCR was carried out on 5 μl of the first round product in a 50 μl reaction using primers CPRF1 (nt 1678–1695, 5′-CAATGTCAACGACCGACC-3′) and CPRR1 (nt 1928–1948, 5′-GAGTAACTCCACAGTAGCTCC-3′), with 5 min hot start followed by 30 cycles of 94 °C for 30 s, 55 °C for 30 s, and 72 °C for 30 s.

For nested PCR of the surface region, first round PCR was carried out in a 50 μl reaction using primers LSOB1 (nt 2739–2762, 5′-GGCATTATTTGCATACCCTTTGG-3′) and P2 [21] with 5 min hot start followed by 30 cycles of 94 °C for 30 s, 50 °C for 30 s, and 72 °C for 90 s. Second round PCR was carried out on 5 μl of the first round products in a 50 μl reaction using primers SSEQ5 (226–246, 5′-AATCCTCACAATACCGCAGAG-3′) and POLSEQ2 (nt 1168–1188, 5′-AGCAAACACTTGGCATAGGC-3′) and the same amplification protocol as first round.

Products from the second rounds were confirmed by agarose gel electrophoresis and then purified using the GenElute™ PCR Clean-up Kit (Sigma, St. Louis MO, USA) according to the manufacturer’s instructions. Cycle sequencing was carried out directly on both strands using 2 μl purified amplicon DNA and primer LSBI1 or ADELN (nt 432–453, 5′-TAGTCCAGAAGAACCAACAAG3′) and a BigDye^®^ Terminator V3.1 Cycle Sequencing Kit (Applied Biosystems, Foster City, CA, USA) according to the manufacturer’s instructions.

### 2.5 Measurement of viral loads

Viral load measurements were carried out as described by Garson et al. [22]. Briefly, HBV DNA was extracted from serum samples using a Qiagen BioRobot 9604 and QIAamp96 Virus Kit reagents (Qiagen, Hilden, Germany). Viral DNA was amplified and quantified in an ABI Prism 7000 sequence detection system (Applied Biosystems, Foster City, CA, USA) using HBV primers and a dual labelled TaqMan probe as described.

### 2.6 HBV genotyping

HBV genotyping were determined using HBV S gene sequences above and the programmes STAR (http://www.vgb.ucl.ac.uk/starn.shtml) [23] and the NCBI Genotyping Tool (http://www.ncbi.nlm.nih.gov/projects/genotyping/formpage.cgi).

### 2.7 Statistical methods

For all analyses, a logarithmic transformation was applied to all viral load measurements prior to analysis to achieve an approximately normal distribution.

#### 2.7.1 Cross-sectional analysis

Unadjusted comparisons between those with and without the BCP mutations were performed using unpaired *t*-tests on the logged values; multiple linear regression analysis was then used to assess whether any differences remained significant after adjusting for HBeAg status and other factors (age, sex and HBV genotype). Formal tests of interaction were performed to determine whether any associations noted differed quantitatively or qualitatively between those who were HBeAg negative and those who were HBeAg positive.

#### 2.7.2 Longitudinal analysis

In the longitudinal analyses, viral loads at 12, 24 and 36 months, and pre- and post-HBeAg seroconversion were summarised using medians and ranges; logged viral loads were then compared in those with and without the double mutations at each timepoint using unpaired *t*-tests. For the group who experienced HBeAg seroconversion, assessment of the significance of changes in viral load from pre- to post-seroconversion, overall and within each subgroup, was performed using a Signed ranks test as the changes, even after logarithmic transformation, were not normally distributed.

All *p*-values were two-tailed, and *p* < 0.05 was considered to be significant.

## 3 Results

### 3.1 Cross-sectional analysis of the association of BCP double mutations and viral loads

Baseline viral loads in the various subgroups are shown in Table 1. Without adjustment for other factors, significantly lower viral loads were seen in those with triple mutations at nt 1753 (T → V), 1762 (A → T) and 1764(G → A) (*p* = 0.03), in those with mutations between nt 1809 and 1817 (*p* = 0.03) and in those with preC stop mutations (nt 1896) (*p* = 0.0001). There was a marginally non-significant trend towards lower viral loads in those with BCP double mutations (1762/1764, *p* = 0.06). Furthermore, viral loads were higher in those who were HBeAg positive (*p* = 0.0001). After controlling for HBeAg status, viral loads remained significantly lower in those with BCP double mutations and preC stop mutations (see Table 2), but the apparent associations with triple mutations (nt 1753, 1762 and 1764) and mutations between nt 1809 and 1817 became non-significant. There were no consistent significant associations between viral load and any of the other parameters considered, including age, sex and viral genotype. Of note, among the individuals analysed, only three had triple mutations at 1762 (A → T), 1764 (G → A) and 1766 (C → T) and two had quadruple mutations at 1753 (T → V), 1762 (A → T), 1764 (G → A) and 1766 (C → T). Thus, the numbers of such individuals are too small to reach any meaningful conclusions about the independent impact of multiple mutations on viral loads.

When the analyses were stratified according to HBeAg status, the association with BCP double mutations appeared to be much stronger in those who were HBeAg positive than those who were HBeAg negative. A formal interaction test (*p* = 0.004) confirmed that the effect of BCP double mutations differed between those who were HBeAg positive and negative. In particular, whilst there was strong evidence of lower viral loads among HBeAg positive individuals with BCP double mutations than those without these mutations, no such association was present among those who were HBeAg negative. In contrast, although the impact of preC mutations on viral loads appeared to differ between those who were HBeAg positive and negative (and were not significant in these two subgroups) there was no significant interaction (*p* = 0.26) between the two factors, suggesting no evidence that the effect differed between the two groups.

### 3.2 Three-year longitudinal analysis of the association of BCP double mutations and viral loads

**Fig. 1:**
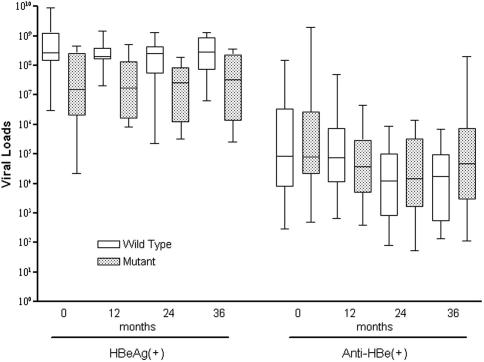
Longitudinal analysis of viral loads according to BCP double mutations and HBeAg status. The box plot shows median values, upper and lower quartiles and the largest and smallest observations.

Of those who were HBeAg positive, 13 subjects with BCP double mutations and 9 without were included in the longitudinal analysis; of those who were HBeAg negative, 17 and 19 subjects were in the two subgroups, respectively. The medians of the viral loads for both the BCP wild-type and BCP double mutations subgroups are higher in the HBeAg positive group than the anti-HBe positive group over 3 years. In the HBeAg positive group, viral loads were significantly lower in the BCP double mutations subgroup than the wild-type subgroup at months 0, 12, 24 and 36 (*p* = 0.02, 0.001, 0.04 and 0.008 at each timepoint, respectively). However, in the anti-HBe positive group, there were no significant differences in the viral loads of the BCP double mutations and BCP wild-type subgroups at the same timepoints (*p* = 0.61, 0.25, 0.61 and 0.12, respectively; Table 3 and Fig. 1).

### 3.3 Association of BCP double mutations and viral loads before and after HBeAg seroconversion in individuals without evolution of the BCP

**Fig. 2:**
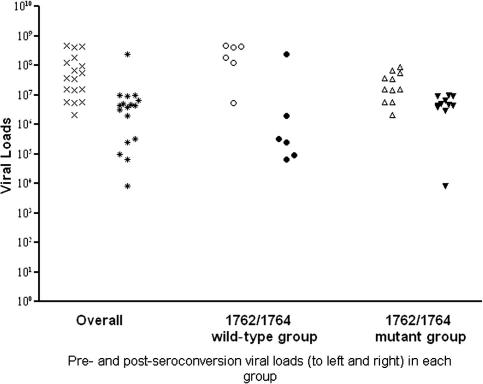
Pre- and post-HBeAg seroconversion vviral loads, stratifies by the presence of BCP double mutations, among 17 individuals without BCP evolution.

Seventeen individuals (11 with BCP double mutations, 6 with wild-type BCP) contributed to the analysis of changes in viral loads before and after HBeAg seroconversion. All samples were taken in July or January of each year; thus, the pre-seroconversion sample would be expected to be on average 3 months prior to seroconversion (although the minimum time from seroconversion would be 1 day and the maximum 6 months) and the post-seroconversion sample would be expected to be taken on average 3 months after seroconversion (with a range of 1 day to 6 months, as above). Overall, there was a significant decrease in viral load from pre- to post-HBeAg seroconversion; whilst this decrease was significant in both subgroups, it was significantly greater in the group that did not have BCP double mutations (*p* = 0.03, Mann-Whitney *U* test). As a result, whilst viral loads were significantly lower prior to HBeAg seroconversion in those with BCP double mutations than those without these mutations (*p* = 0.01), the differences between the two groups were no longer significant after loss of HBeAg (*p* = 0.27) (Fig. 2). This suggests that, whilst BCP double mutations are associated with lower viral loads in HBeAg positive individuals, they have no effect on the viral loads of HBeAg negative individuals.

### 3.4 Association of BCP double mutations and viral loads before and after evolution of BCP double mutations in individuals without HBeAg or anti-HBe seroconversion

Because evolution of the BCP sequence was observed only in a few individuals, the number of individuals with samples available for analysis is too small to reach any reliable conclusions. However, it is clear that all viral loads decline after evolution of the BCP sequence from wild-type to double mutations in individuals who remained HBeAg positive after the mutations were detected. However, viral loads may decline or increase after evolution of BCP mutations in individuals who remained HBeAg negative (Table 4). These results also suggest that BCP double mutations have no effect on viral loads in anti-HBe positive individuals but determination of their effect in HBeAg positive individuals requires further study.

## 4 Discussion

The principal finding of this study is that BCP double mutations are associated with lower viral loads in HBeAg positive individuals but have no effect on the viral loads of anti-HBe positive individuals. Other mutations in the BCP and the major precore mutation at nt 1896 are not associated with viral loads. The strength of this study is that the cross sectional analysis is supported by a long-term longitudinal analysis, which overcomes the deficiencies associated with fluctuations of viral loads, especially around the time of seroconversion of HBeAg. The weakness of the study is the limitation of sample size. Although the double mutations do not prevent the synthesis of HBeAg, they suppress its levels [2,7,8], so that samples with both HBeAg positive and BCP double mutations are uncommon. We do not have sufficient numbers in the cohort for the subgroup of females who are HBeAg positive and have BCP double mutations and this prevented us from including sufficient numbers for the stratification analysis and answering completely all of the questions posed initially. There were very few individuals with evolution of the BCP without HBeAg seroconversion, so that a statistically significant conclusion could not be reached, although the limited number of samples available provides evidence of little effect on viral loads.

Reports of the association of BCP double mutations with virus replication have reached conflicting conclusions: increased virus replication [2,7,8], no effect on virus replication [9–12,15,24] or reduced virus replication [13], and it is not clear which conclusion is reliable. Most of these studies are based on transfecting HBV DNA into cells in culture but virus replication *in vitro* differs from that *in vivo*, where it is influenced by the interaction of virus with the complex host immune system. The effect of BCP double mutations on virus replication *in vitro* may not be representative of the situation *in vivo* [25]. Although other studies are based on clinical samples, they all involve cross-sectional analysis and viral loads may fluctuate over time [19]. In addition, seroconversion from HBeAg to anti-HBe, either spontaneous or after antiviral therapy, usually indicates lower viral loads. This decline may occur up to one year before HBeAg seroconversion [18]. One measurement, or measurements made close to seroconversion of HBeAg, may not be ideal for evaluating the effect of BCP double mutations on viral loads. This study included not only a cross-sectional analysis but also a long-term longitudinal analysis. Therefore, the results are more reliable than previous studies.

The natural course of chronic HBV infection can be divided into four phases based on the virus–host interaction: immune tolerance, immunoreactive, low or non-replication, and reactivation. Virus replication proceeds at very high levels in immune tolerance phase, while HBV DNA levels fluctuate, but decreases progressively in the second, immunoreactive phase. HBeAg remains positive until clearance by the immune system at the end of the second phase. The third phase is characterised by undetectable HBeAg and anti-HBe positivity, with undetectable or low levels of HBV DNA [26]. It has been suggested that BCP double mutations may be selected through CTL escape when immune tolerance is lost and HBeAg concentrations are falling [27]. It may be postulated that, among individuals who are positive for HBeAg, those with wild-type and mutant BCP sequences are in different phases of infection, the former being in the first phase and the latter, at the end of second phase. They experience different immune pressure, resulting in different levels of virus replication. However, those who are HBeAg negative, regardless of BCP sequence, are in the same phase (the third phase) and experience similar immune pressures, resulting in similar levels of virus replication. If this is the case, it is not difficult to understand why BCP double mutations are associated with lower viral loads in HBeAg positive individuals but have no effect in HBeAg negative individuals.

It has been suggested that BCP double mutations are associated with the development of HCC [28–33] and this finding has been confirmed recently by our prospective cohort study [20]. However, the mechanisms of oncogenesis remain obscure. Although we hypothesized originally that BCP double mutations result in an increase in the levels of replicative intermediates and, consequently, of integration events [29], the results of this study do not support this hypothesis and suggest that the association of BCP double mutations with the development of HCC is not attributable to increased viral DNA replication. In addition, it has been postulated that fulminant liver failure may be the result of increased virus replication resulting from the BCP mutations, which may upregulate pregenomic RNA, with concurrent downregulation of precore RNA synthesis [15]. Again, our results do not support this postulate.

The association of other combinations of mutations, such as the triple core promoter mutations 1753/1762/1764, and viral loads remain uncertain. Huang et al. [34] suggested that these triple mutations are associated with lower HBV viral loads but others claim that they increase viral loads [14,15]. We found there is no correlation of this combination of mutations with viral loads. Although mutations at nt 1766 and 1768 have been suggested to be associated with increased virus replication [14,15], these mutations are rare in our cohort (3 in 190 at nt 1766 and none in 190 at nt 1768). Mutations at nucleotide 1809–1817 may be associated with lower HBV viral loads [34] but this is not clear from the present study. A report from Hong Kong show that the major precore stop mutation is associated with lower viral loads [8] while there was no correlation between the presence of the mutation and HBV DNA levels in a Korean study [12]. The data presented here support the latter conclusion.

## Figures and Tables

**Table 1:** HBV core promoter mutations and HBeAg status and viral loads.

Groups	No.	Age^a^	Genotype^b^ (B/C/O/U)	Triple mutation (T1753V)	Triple mutation (C1766T)	Quadruple mutation (T1753V/C1766T)	Mutation (nt 1809–1817)	Precore stop mutation	Viral loads
									Median (range)
**Male**									
*HBeAg*									
1762/1764: Wild-type	23	34.9 ± 6.0	1/14/1/7	0	0	0	0	1	4.3 × 10^8^ (3.0 × 10^6^, 9.0 × 10^9^)
1762/1764: mutant	25	36.4 ± 5.7	0/19/0/6	8	0	0	1	0	2.2 × 10^7^ (3.9 × 10^5^, 3.6 × 10^8^)

*anti-HBe*									
1762/1764: Wild-type	24	40.5 ± 6.2	8/11/0/4	0	0	0	4	13	4.6 × 10^4^ (1.9 × 10^2^, 1.9 × 10^8^)
1762/1764: mutant	26	38.2 ± 6.0	4/19/0/3	13	2	1	4	7	1.6 × 10^5^ (2.7 × 10^3^, 2.0 × 10^9^)

**Female**									
*HBeAg*									
1762/1764: Wild-type	25	34.8 ± 6.0	1/18/0/6	0	0	0	2	1	2.6 × 10^8^ (5.5 × 10^5^, 1.6 × 10^9^)
1762/1764: mutant	18	37.8 ± 5.9	0/17/0/1	5	0	0	1	0	5.6 × 10^7^ (2.2 × 10^4^, 6.8 × 10^8^)

*anti-HBe*									
1762/1764: Wild-type	25	43.8 ± 6.9	9/12/1/3	0	0	0	6	20	1.5 × 10^5^ (6.9 × 10^2^, 4.2 × 10^7^)
1762/1764: mutant	24	42.1 ± 7.1	0/21/1/1	15	1	1	4	11	8.9 × 10^4^ (4.9 × 10^2^, 1.9 × 10^8^)

a Ages are means ± standard deviation.

b B and C: genotypes B and C; O: other genotypes, such genotype A or D; U: undetermined genotype using the current programme.

**Table 2:** Results from multivariable regression analyses.

	Effect of each parameter on the mean viral load (after log transformation)	Standard error	*p*-value
*All study subjects*			
HBeAg positive	2.38	0.21	0.0001
BCP double mutations present	−0.54	0.18	0.003
preC stop mutations present	−0.58	0.24	0.02

*anti-HBe positive (n* *=* *99)*			
BCP double mutations present	−0.01	0.30	0.96
preC stop mutations present	−0.48	0.31	0.12

*HBeAg positive (n* *=* *91)*			
BCP double mutations present	−1.05	0.18	0.0001
preC stop mutations present	0.11	0.60	0.86

**Table 3:** Longitudinal analysis of HBV double mutations and HBeAg status and viral loads.

Groups	No.	Viral loads			
		Month 0	Month 12	Month 24	Month 36
		Median (range)	Median (range)	Median (range)	Median (range)
*HBeAg (+)*					
1762/1764: wild-type	13	2.8 × 10^8^ (3.0 × 10^6^, 9.0 × 10^9^)	2.0 × 10^8^ (2.0 × 10^7^, 1.5 × 10^9^)	2.5 × 10^8^ (2.2 × 10^5^, 1.3 × 10^9^)	2.8 × 10^8^ (6.3 × 10^6^, 1.3 × 10^9^)
1762/1764: mutant	9	1.5 × 10^7^ (2.2 × 10^4^, 4.5 × 10^8^)	1.7 × 10^7^ (8.0 × 10^5^, 5.2 × 10^8^)	2.5 × 10^7^ (3.2 × 10^5^, 1.9 × 10^8^)	3.3 × 10^7^ (2.5 × 10^5^, 3.6 × 10^8^)
*p*-value^a^		0.02	0.001	0.04	0.008

*anti-HBe (+)*					
1762/1764: wild-type	17	8.5 × 10^4^ (2.8 × 10^2^, 1.5 × 10^8^)	7.7 × 10^4^ (6.4 × 10^2^, 5.0 × 10^7^)	1.2 × 10^4^ (7.8 × 10^1^, 8.5 × 10^5^)	1.8 × 10^4^ (1.3 × 10^2^, 6.8 × 10^5^)
1762/1764: mutant	19	8.1 × 10^4^ (4.9 × 10^2^, 2.0 × 10^9^)	3.7 × 10^4^ (3.8 × 10^2^, 4.4 × 10^6^)	1.4 × 10^4^ (5.2 × 10^1^, 1.4 × 10^6^)	4.6 × 10^4^ (1.2 × 10^2^, 2.0 × 10^8^)
*p*-value^a^		0.61	0.25	0.61	0.12

a *p*-Value obtained from unpaired *t*-test after log transformation.

**Table 4:** Evolution of BCP and viral loads, without HBeAg seroconversion.

Samples	Sex	Age	BCP 1^a^	BCP 2^b^	HBeAg 1	HBeAg 2	Viral loads 1	Viral loads 2
GA070	F	30	WT	DM	+	+	2.23 × 10^7^	9.54 × 10^6^
JS19	F	30	WT	DM	+	+	3.27 × 10^8^	2.47 × 10^8^
TJ163	M	36	WT	DM	+	+	3.66 × 10^8^	1.48 × 10^8^
YF163	F	30	WT	DM	+	+	3.80 × 10^8^	1.23 × 10^8^
NX109	M	35	WT	DM	+	+	2.46 × 10^8^	1.60 × 10^8^
CZ041	M	35	WT	DM	–	–	9.18 × 10^5^	1.42 × 10^7^
DD928	F	37	WT	DM	–	–	1.83 × 10^4^	96.55
GM256	M	30	WT	DM	–	–	5.80 × 10^7^	3.29 × 10^8^
TW215	M	45	WT	DM	–	–	2.79 × 10^5^	3.92 × 10^6^
TM334	F	31	WT	DM	–	–	402.61	94.44

a WT: BCP wild-type.

b DM: BCP double mutations.
